# Ambulance Transport of Patients with Mild Conditions in Hokkaido, Japan

**DOI:** 10.3390/ijerph17030919

**Published:** 2020-02-02

**Authors:** Hiroshi Yazaki, Hiroshi Nishiura

**Affiliations:** 1 Graduate School of Medicine, Hokkaido University, Kita 15-Jo Nishi 7-Chome, Kita-ku, Sapporo-shi, Hokkaido 060-8638, Japan; hyazak@gmail.com; 2 Hosanna Family Clinic, Miyanosawa 3 Jo 3-6-1, Nishiku, Sapporo 0630053, Japan

**Keywords:** emergency medicine, epidemiology, dispatch, emergency mobile units, socioeconomic factors, ecological study

## Abstract

Understanding the epidemiological distributions of ambulance transport for patients with mild conditions according to age, disease, and geographic region could help in achieving optimal use of ambulance services. In the present study, we explored the descriptive epidemiology of ambulance transports in Hokkaido, the northernmost prefecture of Japan, identifying potential factors that determine the frequency of transports for mild diseases. Of the total 153,667 ambulance transports in Hokkaido during 2016, we found that two-thirds were for older people, of which about 60% resulted in hospital admission. There were 74,485 transports for mild cases, which were most commonly for psychiatric disorders among working-age adults (n = 4805), heart diseases among older people (n = 4246), and sensory organ diseases among older people (n = 3589). Examining the ecological correlations over 58 geographic units of ambulance services, the total unemployment rate and distance to the nearest tertiary care hospital were, respectively, positively and negatively correlated with the standardized transport ratio for multiple mild diseases. The proportion of working-age adults was uniquely identified as a possible positive predictor in mild cases of psychiatric disorders. As the identified potential predictors could be helpful in considering countermeasures, the causal links should be examined in future studies.

## 1. Introduction

Ambulance dispatch for emergency care in Japan is mostly a public service organized by the Fire and Disaster Management Agency (FDMA), belonging to the Ministry of Internal Affairs and Communications. The service adheres the Fire Service Act, and more than 90% of ambulance calls are made by patients or persons including family members who witnessed patients in need of emergency care. Less than 10% of ambulance calls are triggered by physicians to transport patient between hospitals, but such transport is restricted to patients requiring higher-level emergency care, and a private transport service would be common choice for patients of non-emergency. Out of 18 million patients of emergency care in 2015, 5.3 million (28.9%) used the ambulance transport [1]. To improve the survival rate of emergency patients, ambulances act as one of the most important parts of the social infrastructure, ensuring the health security of individuals and communities. This is even more so in Hokkaido, the largest and northernmost prefecture, covering 22% of the land area of Japan. In 2017, there were a total 6.3 million actual ambulance dispatches across Japan [1], of which acute diseases accounted for 64.3% of all transports, followed by injuries (15.4%), traffic accidents (8.1%), and others (9.4%). An ambulance can reach the patient’s location in 8.6 minutes, on average, which is 1.6 minutes longer than a decade ago. Similarly, the average time lag for an ambulance to reach a hospital is 39.3 minutes, which is 5.9 minutes longer than a decade ago [1].

One of the most well-known factors related to the increase in ambulance calls is aging [1,2,3]. Older people are vulnerable to contracting severe diseases, including brain and neurological diseases and heart disease [4]. However, it has been pointed out that a substantial number of patients with mild conditions may also arise in this age group [5]. Approximately half of ambulance transports have been determined on arrival to be for such mild cases [6], and this proportion has not greatly varied (or improved) in Japan over the past two decades [1]. Unnecessary ambulance use by patients with mild conditions can be regarded as a waste of medical resources and can potentially lead to the congestion of emergency outpatient services and even the loss of the quality in clinical services [5,7,8,9,10].

In Japan, a physician who cares for an emergency patient is mandated to report the classification of disease and its clinical severity upon the first physical examination. The reported data are maintained by the fire department of each locality and are then collected by the FDMA, which prepares an annual official report on the current situation of emergency services [1]. Whereas the epidemiological situation for all of Japan could be grasped using such a reporting system, more detailed information on the characteristics of emergency services according to prefecture, and especially with respect to age, disease, and severity, have yet to be elucidated. Such information is essential to improving emergency services at local level. As the individuals who use emergency services vary geographically and spatially, owing to the different characteristics of disease and different demographic structures, understanding the epidemiological distribution of the above-described mild cases according to age, disease, and severity could help in achieving optimal use of ambulance services in the future [5].

The aims of the present study were to (i) clarify the descriptive epidemiological features of ambulance transport in Hokkaido according to age, geographic region, disease, and severity, especially focusing on mild cases; and (ii) identify the demographic, socioeconomic, and health care-associated factors in ambulance transport of patients with mild conditions.

## 2. Materials and Methods

### 2.1. Study Population

Out of 47 prefectures in Japan, the present study focuses on Hokkaido, the second largest main island and the northenmost prefecture [11]. The population size of Hokkaido is 5.3 million people with the capital Sapporo populated with 1.9 million [11]. It is known that aging has progressed in remote areas, and proportions of working-age (aged from 18 to 64 years) and elderly (aged 65 years or older) were 59.6% and 29.1%, respectively, in 2015 [12], with the dependency ratio estimated at 67.9. The total unemployment rate is 2.9% in 2018. Other than aging and associated population decrease, two important characteristics of Hokkaido are that (i) the ambulance transport system must cover a long distance to the nearest health-care facility and (ii) the great majority of advanced medical care institutes are concentrated in the capital city, Sapporo.

### 2.2. Ambulance Transport Data

Of the total ambulance transports, in the present study, we investigated transports for acute diseases in Hokkaido Prefecture. The ambulance calls for acute diseases were all made by patients or persons who witnessed them. There are 58 regional areas (i.e., geographic units) with fire stations in Hokkaido, corresponding to all ambulance calls within the region [11]. The Department of General Affairs of Hokkaido Prefecture has collected and organized the statistical records of ambulance transports according to region. We analyzed a secondary dataset of the ambulance dispatch record for the period 1 January to 31 December 2016 for all of Hokkaido. The secondary dataset is a yearly snapshot of the distribution of cases by region, age, disease, and severity. Age is summarized according to three discrete groups: minors (up to 17 years old), adults (18–64 years old), and older adults (age 65 years and older). Diseases are classified into eight groups according to organ system: (i) brain and neurological disorders, (ii) heart diseases, (iii) gastrointestinal disorders, (iv) respiratory diseases, (v) psychiatric disorders, (vi) sensory organ disorders, (vii) urinary tract disorders, and (viii) neoplasms. Severity is also classified into four discrete groups: (i) mild, (ii) intermediate, (iii) severe, and (iv) dead on arrival [1], with severity judged by physicians at emergency hospitals. A (i) mild case is defined as a patient who can be managed in the home, (ii) an intermediate case is defined as a patient who may require admission for less than 3 weeks, and (iii) a severe case refers to a patient who requires hospitalization for 3 weeks or more [1]. In the present study, we simplified the severity classifications to mild cases, dead, and the remaining patients. 

### 2.3. Additional Statistical Variables

To explore the underlying mechanisms of ambulance transport, especially among mild cases, we collected additional datasets that represent the demographic dynamics, socioeconomic status, and health care-associated situation in Hokkaido. As for the demographic dynamics, we collected three variables in 58 ambulance regions: (i) population density (retrieved from the population census in 2015 [11]), (ii) the proportion of working-age adults (retrieved from a household survey in 2016 [12]), and (iii) the proportion of one-person households (based on a household survey [11]); all of these factors could potentially lead to increased transports of mild cases. Regarding socioeconomic status, (iv) the rate of public assistance (retrieved from public assistance statistics in 2016 [13]) and (v) total unemployment rate (retrieved from social and demographic statistics [14]) were obtained from census data in the 58 regions. Moreover, we investigated (vi) the estimated distance to the nearest tertiary care hospital, (vii) the number of individuals per physician, and (viii) the number of individuals per hospital bed. Using Google Maps (Google, 2005), the authors measured the distance from the fire department to the nearest tertiary care hospital from each city, town, and village [15]; we subsequently took the weighted average according to the population size of each city, town, and village in each ambulance region [12]. The number of physicians was obtained from a survey of physicians, dentists, and pharmacists [16]. The population size of each ambulance region [12] was divided by the total number of physicians. The number of hospital beds was calculated among secondary care hospitals [17], with the number of beds retrieved from a report of hospital beds of the Hokkaido prefectural government [18]. 

### 2.4. Outcome Measurement and Descriptive Analysis

To compare the rate of ambulance transport in the 58 regions, the rate must be adjusted by the demographic age distribution of the population [12,19]. As the age distribution and population size are heterogeneous by region, we used an indirect standardization method for age, calculating the standardized transport ratio (STR); we used the STR as the primary outcome in this study. In our STR, we regarded Sapporo city, the capital of Hokkaido, as the standard population. An STR greater than 1 indicates a greater number of ambulance transports than Sapporo.

We calculated the STR for all transports regardless of severity, and we also estimated the STR of mild cases according to disease. To understand regional characteristics, the STR was geographically mapped by region for different diseases. To generate the geographic maps, we used the geographic information system MANDARA version 10.0.0.8 (KTGIS.net, Tokyo, 2019) [20]. 

In addition to mapping, we drew the distribution of the number of cases by age, disease, and severity, to clarify their descriptive characteristics. To understand the absolute volume of the impact of ambulance transport, the absolute number of transports was used in this analysis. 

### 2.5. Statistical Analysis

We investigated correlations between the STR and the above-mentioned demographic, sociocultural, and health care-associated variables. We conducted univariate linear regression analysis. Prior to the analysis, the dependent variable (the STR) and a part of the explanatory variables (population density, distance to a tertiary care hospital, persons per physician, and persons per bed) were skewed, and the natural logarithm was taken to approximate their distributions to normal. To address potential confounders, we also implemented multiple linear regression analysis. Only variables that appeared to be significantly correlated during univariate analysis were included. Stepwise regression was conducted with the variable decreasing method and using the minimum Bayesian information criterion to choose the best model. 

### 2.6. Ethical Considerations

We analyzed secondary data that were summarized by the Hokkaido prefectural government. As the data did not contain any identifiable information of individual patients, informed consent was not required for the analyses. The Medical Ethics Committee of Hokkaido University Graduate School of Medicine (Japan) reviewed and approved this study (ID: Med19-052).

## 3. Results

**Figure 1:**
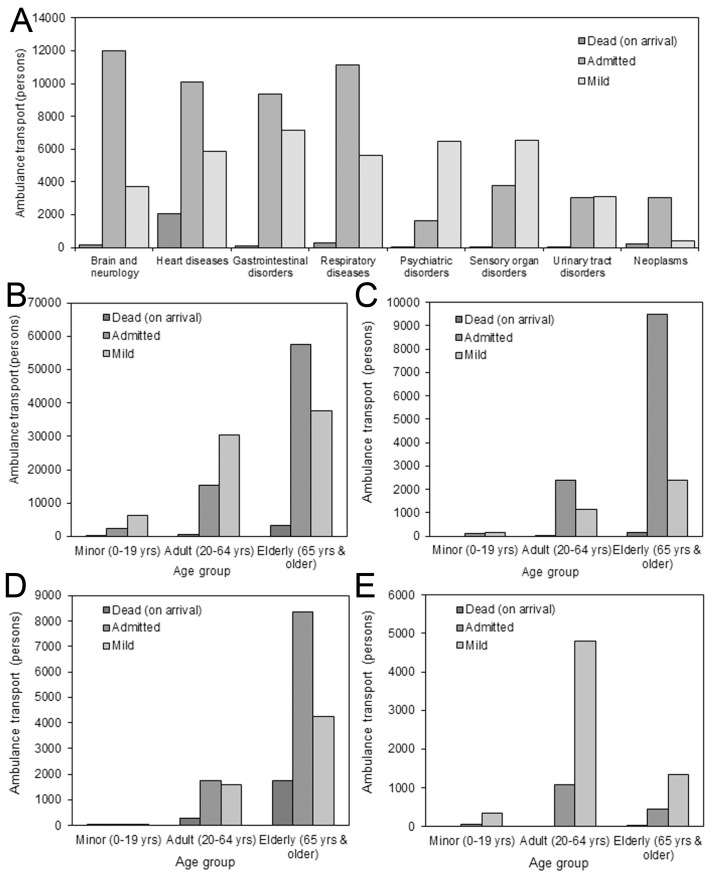
Severity distributions of patients transported by ambulance in 2016. All ambulance transports in Hokkaido during 2016, stratified by severity upon arrival (n=153,667). Disease-specific distributions were further stratified by age group. (**A**) All transports by severity and disease. (**B**) All transports by severity and age group. (**C**) Brain and neurological disorders by severity and age group. (**D**) Heart diseases by severity and age group. (**E**) Psychiatric disorders by severity and age group.

There were a total 153,667 ambulance transports in Hokkaido during 2016, consisting of 3902 (2.5%) transportees who were dead on arrival, 75,280 (49.0%) who were admitted to the hospital, and 74,485 (48.5%) non-admitted transported patients (Figure 1A). Brain and neurological diseases, heart diseases, gastrointestinal disorders, and respiratory diseases were the four leading reasons for ambulance transport, accounting for 44.0% of total transports. The three most frequent disease categories among admitted patients were brain and neurological diseases (n = 11,995; 15.9%), respiratory diseases (n = 11,107; 14.8%), and heart diseases (n = 10,096; 13.4%); the three most frequent diseases among non-admitted patients were gastrointestinal disorders (n = 7149; 9.6%), sensory organ disorders (n = 6537; 8.8%), and psychiatric disorders (n = 6481; 8.7%). 

Figure 1B–D shows a comparison of the distributions by age and severity. Of total ambulance transports, minors, adults, and older people accounted for 8744, 46,299, and 98,624 transports, respectively, with older people representing 64.2% of the total. Among all admitted transportees, older people accounted for 57,517 (76.4%). Focusing on mild and non-admitted cases (Figure 1B), minors, adults, and older people accounted for 6315, 30,405, and 37,765 patients, respectively, with each representing 72.2%, 65.7%, and 38.3% of total transports within the same age group, respectively. Individuals with brain and neurological diseases were very likely to be admitted (75.6%); of these, older people accounted for 79.3% (Figure 1C). Dead on arrival was seen in 11.3% of transportees with heart diseases, among which 86.2% were older people (Figure 1D). In total, 80.2% of individuals with psychiatric disorders were not admitted, among which 74.1% were working-age adults (Figure 1E). The most frequently seen combination by age group and disease for mild cases was psychiatric disorders in working-age adults (n = 4805) followed by heart diseases among older people (n = 4246) and sensory organ diseases among older people (n = 3589). 

**Figure 2:**
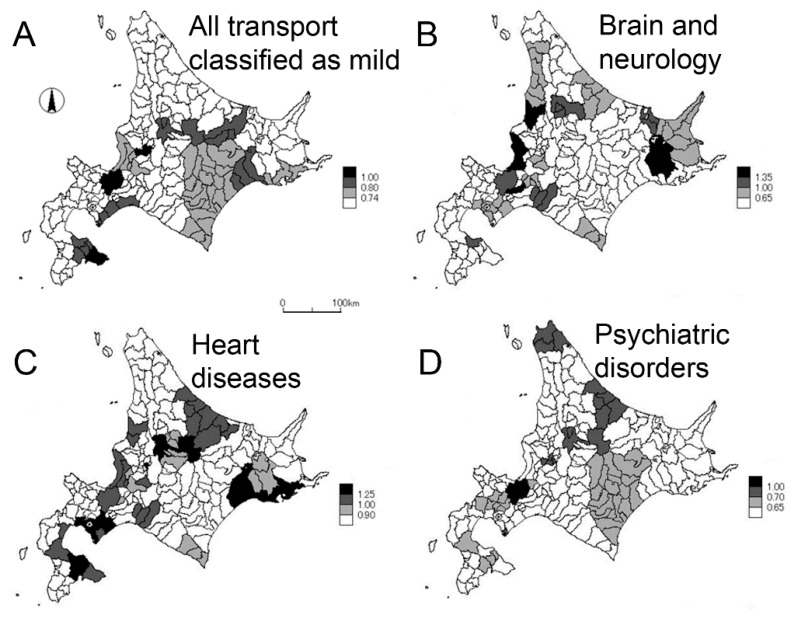
Geographic distributions of the standardized transport ratio for mild diseases, according to administrative district of emergency services. The standardized transport ratio for mild diseases upon arrival was calculated using Sapporo, the capital city, as the baseline, adjusting for the age distributions. Four discrete categories were defined and scaled (lower right of each panel). (**A**) All diseases, (**B**) brain and neurological disorders, (**C**) heart diseases, and (**D**) psychiatric disorders.

Figure 2 shows the geographic distributions of the STR in mild cases for all transports classified as mild, brain and neurological diseases, heart diseases, and psychiatric disorders. For all mild conditions, brain and neurological diseases, and heart diseases, the STR tended to be higher in urban areas, including the three most populated cities: Sapporo, Asahikawa, and Hakodate (Figure 2A–C). Other than urban cities, brain and neurological diseases were common in eastern Hokkaido (Figure 2B), whereas heart diseases were more frequent in southern Hokkaido (Figure 2C). Psychiatric diseases were also more frequently seen in urban cities including Sapporo and Asahikawa, but the STR of mild cases was also high in northern Hokkaido (Figure 2D).

**Figure 3:**
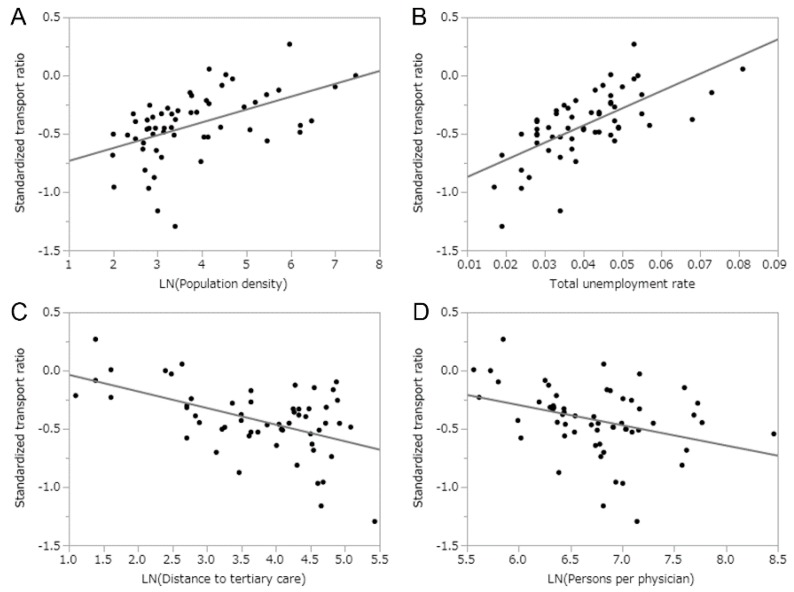
Univariate relationships between the standardized transport ratio for mild diseases upon arrival and socioeconomic characteristics. Scatter plots show the correlations between two variables. Horizontal axes are (**A**) population density, (**B**) total unemployment rate, (**C**) distance to the nearest tertiary care hospital, and (**D**) population per physician. Lines represent the best-fit prediction based on univariate linear correlation analysis. It should be noted that logarithmic scaling was used for the vertical axes and also the horizontal axes of A, C, and D.

**Figure 4:**
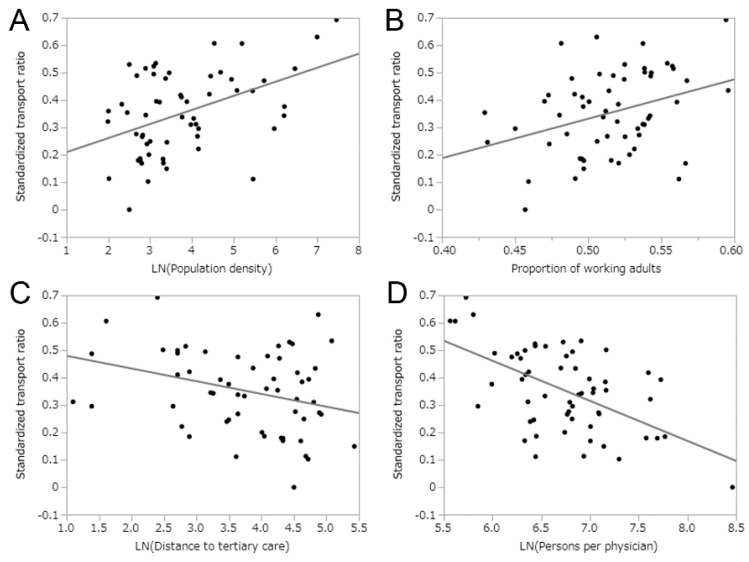
Univariate relationships between the standardized transport ratio for mild psychiatric diseases upon arrival and socioeconomic characteristics. Scatter plots show the correlations between two variables. Horizontal axes are (**A**) population density, (**B**) proportion of working-age adults in the population, (**C**) distance to the nearest tertiary care hospital, and (**D**) population per physician. Lines represent the best-fit prediction based on univariate linear correlation analysis. It should be noted that logarithmic scaling was used for the vertical axes and also the horizontal axes of A, C, and D.

Table 1 summarizes the results of univariate linear regression for eight diseases plus all transports as well as all transports for mild cases, examining their correlations with demographic, socioeconomic, and health care-associated variables. All transports for mild cases were positively correlated with population density (r = 0.50, *p* < 0.01), rate of public assistance (r = 0.41, *p* < 0.01), and total unemployment rate (r = 0.66, *p* < 0.01), and they were negatively correlated with the distance to tertiary care (r = −0.52, *p* < 0.01) and persons per physician (r = −0.35, *p* < 0.01). Of eight explanatory variables, the population density and total unemployment rate showed significant positive correlations for seven disease categories of the total 10 disease classifications, including all transports and all transports for mild conditions. For instance, the total unemployment rate revealed significant positive correlations with all transports (r = 0.66, *p* < 0.01), all transports for mild cases (r = 0.66 *p* < 0.01), heart diseases (r = 0.51, *p* = 0.01), gastrointestinal disorders (r = 0.43, *p* < 0.01), respiratory diseases (r = 0.48, *p* < 0.01), psychiatric disorders (r = 0.31, *p* = 0.02), and urinary tract disorders (r = 0.50, *p* < 0.01). The proportion of working-age adults was positively correlated with psychiatric disorders (r = 0.34, *p* < 0.01) and neoplasms (r = 0.46, *p* < 0.01). The distance to a tertiary care hospital was negatively correlated with six diseases: all transports (r = −0.50, *p* < 0.01), all transports for mild cases (r = −0.52, *p* < 0.01), gastrointestinal disorders (r = −0.33, *p* = 0.01), respiratory diseases (r = −0.40, *p* < 0.01), psychiatric disorders (r = −0.32, *p* = 0.01), and urinary tract disorders (r = −0.49, *p* < 0.01). Persons per physician was negatively correlated with all transports for mild diseases (r = −0.35, *p* = 0.01), respiratory diseases (r = −0.42, *p* < 0.01), and psychiatric disorders (r = −0.56, *p* < 0.01). No diseases were correlated with the proportion of one-person households and persons per hospital bed. Figure 3 shows correlations between the STR in all transports for mild cases and explanatory variables. The total employment rate showed the strongest positive correlation (r = 0.66). Figure 4 shows the correlation between the STR of mild cases with psychiatric disorders and explanatory variables. Mild psychiatric disorders distinguished themselves from others in that a strong correlation was observed with the proportion of working-age adults (r = 0.34, *p* < 0.01).

Table 2 summarizes the final models in multiple linear regression using a stepwise method for variable selection. The total unemployment rate, distance to tertiary care, and proportion of working-age adults were the most frequently included variables in the final models. Total unemployment rate was positively correlated with all transports (r = 6.31, *p* < 0.01), all transports for mild conditions (r = 12.45, *p* < 0.01), heart diseases (r = 15.49, *p* < 0.01), respiratory diseases (r = 13.50, *p* < 0.01), psychiatric disorders (r = 2.99, *p* = 0.03), and urinary tract disorders (r = 16.58, *p* < 0.01). Distance to tertiary care was negatively correlated with all transports (r = −0.06, *p* < 0.01), all transports for mild cases (r = −0.10, *p* < 0.01), and urinary tract disorders (r = −0.19, *p* < 0.01). As seen in the univariate results, the proportion of working-age adults was positively correlated with psychiatric disorders (r = 1.21, *p* = 0.01) and neoplasms (r = 3.77, *p* < 0.01). Population density was positively correlated with seven diseases in univariate analyses but was eliminated in all of the final models. Brain and neurological diseases and sensory organ disorders showed no significant correlations with explanatory variables in the univariate analysis; this was also the case during multivariate analysis.

## 4. Discussion

The present study explored the descriptive epidemiology of ambulance transports for acute diseases in Hokkaido, the northernmost prefecture of Japan. We showed that two-thirds of ambulance transports were for older people, of which about 60% resulted in hospital admission. The main diseases among transportees included brain and neurological disorders, heart diseases, and respiratory diseases, echoing with the fact that cerebrovascular diseases, ischemic heart disease, and pneumonia are common among older adults, and these are frequently exacerbated [3]. Mild diseases included psychiatric disorders among adults and sensory organ disorders among older people. Psychiatric emergency cases mainly involve alcohol or drug-related disorders, panic and stress disorders, emotional disorders, self-injury, and schizophrenia [21], and the chief complaint of ambulance calls include acute alcoholism (38.8% of the total), hyperventilation (15.1%), self-injury (9.8%), chest discomfort (7.2%), and mood disorder (6.2%) in Kitakyushu, Japan [22]. Sensory organ disorders were represented by vertigo, which is known to cause acute symptoms, although the symptoms are reversible. It has been well documented that these diseases account for a substantial proportion of ambulance transports for mild conditions [6,9]. Geographic distributions of mild cases also differed according to disease. The diseases examined shared the common feature that the high STR values were found in Sapporo, the capital city; however, psychiatric disorders were more common in areas with a greater proportion of working-age adults than in other areas. The STR of heart diseases was higher where the total unemployment rate was higher. The total unemployment rate and distance to tertiary care were frequently included as part of the final model in predicting STR of all transports and transports for mild cases of various diseases. The proportion of working-age adults was positively correlated with psychiatric disorders and neoplasms. 

The present study has three key findings. First, ambulance calls are known to be associated with economic factors. People with lower annual income tend to more frequently use ambulance services in the United States [23] and Canada [24]. Kawakami et al. [25] showed that increased annual income in Japan is associated with fewer calls for ambulance dispatch. Emergency services are more frequently used by individuals with lower socioeconomic status [26] or those without health insurance [2] than other groups. The present study additionally showed that unemployed people may frequently use ambulance transport for mild diseases that do not require hospital admission. Rates of public assistance were not correlated with transports for mild cases, although receiving public assistance was associated with an overall increase in ambulance transports. Such type of health insurance was not associated with ambulance call, perhaps reflecting the background of universal health coverage as the basis of health-care system in Japan, which does not refute patients in need of care. Second, the distance to a tertiary care hospital was shown as a predictor of ambulance transports in patients with mild diseases. This finding may be somewhat controversial because such patients do not require tertiary care. We believe that this finding was caused by a confounder, e.g., the distance to a tertiary care hospital may have reflected the distance to an urban area because tertiary care hospitals are only located in large cities. In fact, the geographic distributions showed that high STRs for mild cases were commonly seen in the three largest cities. Third, mild cases included psychiatric disorders and sensory organ disorders; the former has been shown to be correlated with the proportion of working-age adults in the population. That is, there would be a greater frequency of ambulance transports for psychiatric illnesses in areas where there are more working-age adults.

What are the practical implications of these three findings? First, based on our subprefectural level analysis, unemployment was shown as a factor in ambulance transport for mild cases. Inequality of working conditions in Hokkaido, e.g., the tertiary sector in urban areas with high heterogeneity of working conditions and the primary sector of industry in remote areas, could result in different levels of employment. The improvement of ambulance transport services should be tied to social development, with improved employment conditions. Second, considering that a closer distance to a city might lead to a greater number of transports for mild conditions, possible remedies to this situation might include geographically equalizing the access to emergency care across Hokkaido, for example, providing better access to emergency medicine for people in remote areas. Third, it might be unavoidable to have a greater number of transports for psychiatric disorders in areas where there are more working-age people. However, a possible remedy to both our second and third findings described above is to strengthen emergency triage during ambulance calls in urban areas. In fact, an epidemiological study of psychiatric emergency call has indicated that the majority of cases has been mild in any category of psychiatric illnesses, and a well-designed prehospital triage can reduce the overall risk of psychiatric emergency service [22]. Moreover, psychiatric emergency services for mild conditions could be strategically offered more in urban areas to ensure that people who truly require emergency care appropriately use ambulance transport services. Improving the level of patient education would also be the key to appropriately communicate the need of emergency care using ambulance transport.

In addition, it is worthwhile to discuss negative correlations between the transport of mild cases and persons per physician. Not only the overall transport of mild cases but also mild cases of respiratory diseases and psychiatric disorders yielded significant negative correlations. It is difficult to fully explain the underlying mechanisms, but at least this finding implies a possible impact of excessive provision of medical service on ambulance calls in geographic areas with a substantial density of physicians.

An important remark is that the present study explored statistical correlations. It is well known that correlation alone does not indicate a causal relationship. Thus, the above-mentioned potential interventions may be regarded as overstatement. Improved insights into public health practice could be gained by longitudinal observation of individuals, e.g., examining the relationship between socioeconomic factors and disease risks over time at the individual level, and also by implementing quasi-experimental studies, if possible [27]. 

Several limitations of the present study must be discussed. First, the present study design was an ecological study, which is known to be prone to confounding. Thus, the level of evidence is low, and our findings could have captured what could also be explained by potential confounders. Studies using individual data must be considered in order to strengthen our findings. Second, the results of severity assessment can sometimes change during the course of emergency service. For instance, a patient with a major complaint of vomiting can sometimes appear to be a case of acute myocardial infarction. We relied on judgment based on the initial assessment by a physician. Third, when an ambulance crossed the border between geographic regions, our datasets counted the corresponding cases based on the geographic region of the hospital. The frequency of such cross-border transport is not very common, except in geographic areas very close to the capital of Sapporo; however, it should be remembered that such cases made it difficult to precisely elucidate the correlations between STR and demographic and socioeconomic factors using the ecological study design. Fourth, Hokkaido is the largest prefecture in Japan. When an ambulance cannot reach patients at extremely distant locations who truly require emergency care, helicopter and aircraft transport are sometimes used instead. Thus, our analysis in geographically most distant areas may not be very accurate. 

Although a number of limitations exist, we believe that the present study successfully characterized the descriptive characteristics of ambulance transports of patients with mild conditions in Hokkaido, identifying that the total unemployment rate, distance to tertiary care, and proportion of working-age adults are possible explanatory variables. Verifying these findings in additional studies, including those based on longitudinal or quasi-experimental study designs, can be helpful in developing possible countermeasures to inappropriate use of ambulance transport services.

## 5. Conclusions

In the present study, we explored the descriptive epidemiology of ambulance transports for acute diseases in Hokkaido, identifying potential factors that determine the frequency of transports for patients with mild diseases. We found that two-thirds of ambulance transports were for older people, of which about 60% resulted in hospital admission. Mild diseases included psychiatric disorders among adults and sensory organ disorders among older people. The total unemployment rate, distance to tertiary care, and proportion of working-age adults were identified as possible explanatory variables for the transport of mild cases, the causal links of which should be examined in future studies. As possible countermeasures, (i) improvement of ambulance transport services should be tied to social development, with improved employment conditions, (ii) it is critical to geographically equalize the access to emergency care across Hokkaido, for example, providing better access to emergency medicine for people in remote areas, and (iii) it is vital to strengthen emergency triage during ambulance calls in urban areas.

## Figures and Tables

**Table 1:** Univariate relationship between the standardized transport ratio and sociodemographic/sociocultural variables.

	All Transport and Severity	All Transport Classified As Mild	Brain and Neurology	Heart Diseases	Gastrointestinal Disorders	Respiratory Diseases	Psychiatric Disorders	Sensory Organ Disorders	Urinary Tract Disorders	Neoplasms
Population density ^†^	**0.42 (<0.01) ^‡^**	**0.50 (<0.01) ^‡^**	0.04 (0.74)	**0.33 (0.01) ^‡^**	**0.40 (<0.01) ^‡^**	0.44 (<0.01) ^‡^	0.45 (<0.01) ^‡^	0.01 (0.95)	0.41 (<0.01) ^‡^	0.25 (0.06)
Proportion of working-age adults	0.00 (0.98)	0.10 (0.45)	−0.11 (0.41)	0.02 (0.89)	0.17 (0.20)	0.07 (0.62)	**0.34 (<0.01) ^‡^**	−0.00 (0.98)	0.12 (0.36)	**0.46 (<0.01) ^‡^**
Rate of public assistance	**0.46 (<0.01) ^‡^**	**0.41 (<0.01) ^‡^**	0.02 (0.86)	**0.32 (0.02) ^‡^**	0.25 (0.06)	0.26 (0.05)	0.21 (0.11)	0.18 (0.18)	0.12 (0.38)	0.10 (0.45)
Total unemployment rate	**0.66 (<0.01) ^‡^**	**0.66 (<0.01) ^‡^**	0.13 (0.35)	**0.51 (<0.01) ^‡^**	**0.43 (<0.01) ^‡^**	**0.48 (<0.01) ^‡^**	**0.31 (0.02) ^‡^**	0.23 (0.09)	**0.50 (<0.01) ^‡^**	0.09 (0.50)
Proportion of one-person household	−0.01 (0.94)	0.02 (0.91)	−0.19 (0.16)	−0.02 (0.87)	−0.13 (0.32)	0.13 (0.32)	0.13 (0.34)	−0.01 (0.95)	−0.19 (0.16)	−0.14 (0.28)
Distance to tertiary care ^†^	**-0.50 (<0.01) ^‡^**	**-0.52 (<0.01) ^‡^**	−0.11 (0.41)	−0.24 (0.07)	**-0.33 (0.01) ^‡^**	**-0.40 (<0.01) ^‡^**	**-0.32 (0.01) ^‡^**	−0.16 (0.23)	**-0.49 (<0.01) ^‡^**	−0.15 (0.26)
Persons per physician ^†^	−0.24 (0.06)	**-0.35 (0.01) ^‡^**	0.02 (0.88)	−0.09 (0.50)	−0.20 (0.13)	**-0.42 (<0.01) ^‡^**	**-0.56 (<0.01) ^‡^**	0.02 (0.89)	−0.22 (0.11)	−0.19 (0.15)
Persons per hospital bed ^†^	−0.16 (0.22)	−0.13 (0.33)	−0.01 (0.93)	−0.13 (0.33)	−0.08 (0.56)	−0.06 (0.65)	0.15 (0.25)	−0.19 (0.15)	−0.02 (0.89)	0.19 (0.15)

Correlation coefficient of univariate linear regression is shown. p-values are in parenthesis. † Logarithm was taken because of a skewed distribution. **‡** Significant correlation, with *p* < 0.05.

**Table 2:** Multivariate liner regression models: socioeconomic and health care-associated variables remaining in the final model and correlation with the standardized transport ratio.

	Variables	Correlation Coefficient	p-Value
All transport and severity	Rate of public assistance	2.67	0.12
(R^2^=0.54)	Total unemployment rate	6.31	<0.01
	Distance to tertiary care ^†^	−0.06	<0.01
All transport classified as mild	Total unemployment rate	12.45	<0.01
(R^2^=0.53)	Distance to tertiary care ^†^	−0.10	<0.01
Heart Disease	Total unemployment rate	15.49	<0.01
(R^2^=0.25)			
Gastrointestinal Disorders	Population density ^†^	0.07	0.23
(R^2^=0.21)	Total unemployment rate	11.17	0.06
	Distance to tertiary care ^†^	−0.09	0.18
Respiratory Diseases	Total unemployment rate	13.50	<0.01
(R^2^=0.31)	Distance to tertiary care ^†^	−0.09	0.15
	Persons per physician ^†^	−0.20	0.06
Psychiatric disorders	Proportion of working adults	1.21	0.01
(R^2^=0.38)	Total unemployment rate	2.99	0.03
	Persons per physician ^†^	−0.11	<0.01
Urinary tract disorders	Total unemployment rate	16.58	<0.01
(R^2^=0.36)	Distance to tertiary care ^†^	−0.19	<0.01
Neoplasms	Proportion of working adults	3.77	<0.01
(R^2^=0.32)			

† Logarithm was taken because of skewed distribution. R^2^ represents the adjusted coefficient of determination. Brain and neurological diseases and sensory organ disorders did not reveal any significant correlation during univariate analysis and are therefore not included.
